# Law and Rhetoric: Critical Possibilities

**DOI:** 10.1111/jols.12156

**Published:** 2019-05-20

**Authors:** John Harrington, Lucy Series, Alexander Ruck‐Keene

**Affiliations:** ^1^ School of Law and Politics Cardiff University Cardiff CF10 3AX Wales; ^2^ 39 Essex Chambers London WC2A 1DD England

## Abstract

What contribution can rhetoric make to socio‐legal studies? Though now a byword for deception and spin, rhetoric was long identified with the very substance of law and politics. Latterly radical scholars have foregrounded an understanding of law as rhetoric in their polemics against legal formalism, but it needs to be complemented by a critical perspective which goes beyond simple revivalism, taking account of rhetoric's own blind spots, inquiring into the means by which some speakers and listeners are privileged and others excluded or silenced. The critical potential of legal rhetoric is tested here through a review of the developing law on mental capacity and the best interests of people with disabilities in England and Wales. Much of what is at stake there is properly grasped in terms of a politics of speech: who is addressed, who can speak, who must speak, and how are they represented in judicial and media discourse.

## INTRODUCTION

Nowadays ‘rhetoric’ is a term of abuse in legal scholarship as in wider political discourse. A recent survey of law journal articles suggests that it is usually counterposed, negatively, with the term ‘reality’.^1^ Rhetoric is anti‐fact: mere ornament, lies and spin. Opponents of contemporary political populism, for instance, impugn the idiom as much as the substance of neonationalism in the United States, Britain, and continental Europe. For them, rhetoric describes what comes out of the heated chambers of social media, the late‐night tweets of demagogues. This scepticism and anxiety has accompanied rhetoric since its origins in ancient Greece. It is the burden of Plato's criticisms in the *Gorgias*. A tool crafted by amoral technicians and put at the service of rabble rousers, rhetoric was no more than a ‘flattery’ of logic.^2^

What does it mean to study of law rhetorically? Let's start with a distinction: between rhetorical technique and rhetorical criticism. The former chiefly includes tactics for effective persuasion – a guide, a toolbox, a crib for effective verbal performance: *How Aristotle can Help you to Win Friends and Influence People* or *The Best Man's Duties*. All this has its place in training for advocacy and public speaking, but, as socio‐legal scholars, it need not detain us too long. The latter is our sole focus here. Standing back from verbal tactics, rhetorical criticism involves inquiry into the textual and contextual preconditions for persuasive speech. Criticism has two senses here: the informed and attentive reading of legal materials, as in literary or cultural studies; but also, the engaged study of how social and political forms are produced, reproduced, modified, and challenged in the substance of legal speech and legal writing. To emphasize: both modes are closely intertwined. Style, for instance, needs to be grasped as regards its formal properties, but also politically with a view to how it sustains inequalities of voice and power.

In the following sections we consider the origins, decline, and revival of rhetoric generally and in law in particular. We argue that classical rhetoric still offers useful tools to socio‐legal scholars for reading the output of courts, lawmakers, citizens, and legal activists. We note the influential turn to classical rhetoric in the United States in the early twentieth century and the limited purposes of that retrieval, purposes which were undermined by the emergence of a much more wide‐ranging critical rhetoric in the 1960s. The critical challenge points to the political exclusions of classical rhetoric, in terms of race, class, and gender. It suggests moreover a wider circle of political concern and an augmented analytical repertoire for studying contemporary speech in law and related domains. We then gather together the insights of classical and critical rhetoric, deploying each in order to illuminate struggles over speech and representation in the area of mental capacity law. Ethical and legal debates concerning individual autonomy, medical paternalism, and judicial power, resolve into fundamentally rhetorical questions over who speaks and how are they represented, who is addressed and who silenced. In sum we hope to show that critical rhetorical analysis offers a mode of accounting for law as politics, including its relation to wider social and cultural contexts, which takes the agency of legal actor seriously and which pays due attention to the provisional and creative nature of legal argumentation.

## REPUDIATING AND RECOVERING

The study of rhetoric in Europe dates back to classical Greece, with Aristotle's *Art of Rhetoric* being a key text, and to Roman authors such as Cicero and Quintilian.^3^ Classical rhetoric was a staple of education for lawyers and other professionals in Europe until the rise of rationalism in the seventeenth and eighteenth century.^4^ From this period, it fell into decay, being increasingly cast as a literary supplement to empiricist and rationalist philosophy. Writers from Thomas Hobbes and Jeremy Bentham to John Rawls defined their work as against rhetoric, offering instead rational theories of sovereignty, legislation, political morality, and so on.^5^ The attractions of their theses would lie in indisputable empirical data regarding human nature and society, or in the logical coherence of their a priori schemes and thought experiments. By contrast, as John Locke had it, eloquence and figurative speech served no other purpose than ‘to insinuate wrong ideas, move the passions and thereby mislead the judgment, and so are perfect cheats.’^6^ Modern law and legal theory, in most of their dominant forms, have also participated in this embrace of rationalism and in the repudiation of their own traditions. The perceived amorality of rhetoric put it at odds with natural law thought which sought to anchor legal reasoning, somehow, in a higher order. Its provisional, situation‐bound quality militated against the systemic order and purity valued by codifiers and textbook writers. Romantic opposition to literary rhetoric as artificial, technical, and purely ornamental, registered even more in the case of law, where these attributes suggested a lack of seriousness and an avoidance of profound, core issues.^7^ By turns plural, playful, and polemical, it threatened to disrupt the sober speech of the law.^8^

There are two sources for this repudiation, according to James Boyd White: the Christian tradition in which law is a set of authoritative commands; and modernist sociology which sees it as a system of institutionally established and managed rules.^9^ Taken in combination they presuppose an instrumental view of law with little room left over for rhetoric, which lives on only as the other of scientific legal studies. The law in this mode is ideally a system of rules which are pre‐given and whose meaning is to be found through exegesis rather than actively created in the process of argumentation.^10^ Legal scholarship specializes in this task, promoting the unity of the system through correcting errors and integrating new developments in doctrine. In so far as it draws on other disciplines, it favours those which offer determinate, exclusive answers, for example, neo‐classical economics, and spurns those associated with openness and complexity, for example, literary studies.^11^ Legal theory serves similar ends by sharply delimiting the ‘province of jurisprudence’ (John Austin) or elaborating a ‘pure theory of law’ (Hans Kelsen).^12^ Of course, in a familiar irony, this denial of rhetoric is itself an obviously rhetorical move. Law performs its own authority and self‐sufficiency in text and speech through a neutral style and a coercive idiom (‘it cannot be denied’, ‘no one can doubt’, and so on).^13^ In aiming at closure it rations speech, dominating or marginalizing non‐legal languages and those who bear them. Legal education involves above all imparting this stylistic repertoire and the hierarchy of voice which goes with it, embodied traits which are retained long after the substantive matter of the syllabus has been forgotten.^14^

Rhetoric takes ideas and culture seriously as integral to social practices, including law. As such it provides an antidote to those positivistic social sciences which attend to the regularities in phenomena and in relations between them in a bid to ground themselves and to display their reliability and wider utility.^15^ For such disciplines, ideas are often merely super‐structural, concealing deeper processes or hiding preformed objective interests. By contrast, ideas are taken seriously as social forces by Critical Discourse Analysis (CDA), perhaps the most important neighbouring field to rhetoric.^16^ In its investigation of how cultural forms represent, conceal, and reinforce power inequalities CDA can contribute a great deal to rhetorical analysis. But it is marked by a certain structuralism, which sees blocs of ideas shaping political (and by extension legal) developments on a fairly large scale over longer time periods. For rhetoric, by contrast, ideas, ideologies, and discursive forms exist primarily in actual use, that is, in specific moments of speech or in the production, reception, and appropriation of texts.^17^ In other words, they enjoy a distinct and specifically political reality as they are deployed in argumentation. As Quentin Skinner has shown, ideas live historically, gaining meaning from their strategic articulation in particular social and cultural contexts.^18^ Their contribution to power constellations is, thus, iterative and episodic, rather than simply structural. By contrast with CDA, therefore, the scale of rhetorical analysis is considerably more focused spatially and temporally, concentrating on the timeliness (*kairos*) of a specific utterance or chain of utterances and their appropriateness to a concrete problem (*stasis*).^19^ CDA does not exhaust the field, of course. For example, the particularistic focus of rhetoric is shared by critical discursive psychology. This seeks:

not so much to analyse the changes in society's ‘large‐scale discourses’, which concrete language use can bring about, as to investigate how people use the available discourses flexibly in creating and negotiating representations of the world and identities^20^

in small scale social interaction and to reflect on the consequences of this.

Classical rhetoric provides a well‐stocked toolbox of distinctions for analysing persuasive effects. These include the occasions of speech (that is, forensic, deliberative, and commemorative); the canons of rhetoric (discovery, arrangement, style, delivery, and memory); figures of speech (metaphor, metonymy, synecdoche, and so on); and parts of speech (introduction, narration, proof, refutation, and conclusion). All can aid in the close reading of legal materials, and readers are referred to the significant technical literature on them.^21^ For present purposes it is most useful to concentrate on the three modes of proof in rhetoric – *logos, pathos*, and *ethos* – and the manner in which they shape legal argumentation.^22^ *Logos* refers to the truth, consistency, and plausibility of the argument itself. Its most important feature, for present purposes, is the topic. These common‐sense assumptions and distinctions shared between speaker and audience, are almost literally ‘places’ (*topoi*) from which an argument can begin. They provide a set of pigeonholes (or ‘topic grid’) for categorizing issues and phenomena. Topics may be general, held by all audiences, or particular to specific disciplines. As Jack Balkin has argued, law is constituted by a set of heterogeneous topics, including formal materials, such as precedent cases, statutes, rules of statutory construction, and so on, as well as maxims, standards, principles, and policies, but also presumptions about the natural world and the society in which the law operates.^23^ Topics are not logically coherent and cannot compel a unique conclusion. Rather, they are articulated and combined to make a stronger or weaker case. The may be removed by repeal or overruling, but are more likely to fade in and out depending on the extent to which they are used, especially in common law systems.

*Pathos* involves the evocation of emotions, such as pity, envy, and rage, in order to gain the approval of the audience for a particular conclusion. This may be directed towards the speaker herself or a third party referred to in the speech. Pathos should not be confused with purely subjective emotion. Like logos it has an important public aspect, drawing on shared feelings and stock responses which are sustained by a common visual culture.^24^ It is not common in judicial rhetoric, which tends to cultivate an air of objectivity as we have mentioned. Argumentation in controversial medical cases is one exception to this, as is criminal defence. By contrast, *ethos* is central to the persuasive strategies of judges, advocates, and expert witnesses. Emphasizing the speaker's authority, it is implicit in the distinctive decoration of courtrooms, as well as in the citation practices and modes of address adopted within them. Peter Goodrich has shown how the many ostensibly redundant usages in legal speech and the common tendency to limit the competence of the forum (for example, ‘this court is a court of law, not of morals’) are instances of ethos.^25^ Novel cases, which show the limits of well‐established topics may lead to more forthright assertions of authority. But the three modes of proof will usually be combined to some degree. As Roland Barthes put it, while she speaks the orator must also keep saying, ‘follow me … esteem me … love me’.^26^

## PERFORMING AND PARTICIPATING

This focus on the particular makes rhetoric suitable as a means of taking seriously the interdependent cultural and political nature of law. We can follow Luhmann, for these purposes, in understanding law as a series of discrete textual and verbal episodes recursively linked to other such moments by the requirement to show legal validity in distinguishing lawful from unlawful.^27^ Thus, for example, pleadings for a case and the decision of a court itself, are all connected by chains of normative reference to previous such instances, including precedents, statutory enactments, and binding executive decrees. Each moment can be studied as an attempt to persuade a range of audiences of the legal soundness and factual appropriateness of the outcome and, more broadly, that the normative materials require this conclusion to be reached.^28^ Rhetorical criticism draws us into the particular time and place of these moments of persuasion. It encourages us to take seriously the contingency of the outcome, the crafting of arguments, and the pressure of cultural and social forces upon them. As well as the scholar, the law teacher and her students can only benefit from opening‐up inquiry in this way.^29^ Rhetoric outflanks orthodox doctrinal analysis which, as we know, cultivates a certain blindness as to the identity of the speaker and as to the constitution and location of her audience, and which aims to condense the actual words of the judge or parliamentarian into a kernel of rules and principles, with much of what was actually said cast off as mere interpretive chaff.^30^ Agency, creativity, and chance are given their due as they are not by legal formalism and structuralist social theory.^31^ Law is performance.^32^ It is something we do, not something which we have as a result of what we do.^33^

This understanding of law as argument and performance in the first instance also points to the manner in which rhetoric indexes law's politics. Let's go back to Plato. For him government of the polis was most appropriately subject to incontestable principles, not localized argument and contingent decision making.^34^ As Jacques Rancière put it, Plato proposed an ‘archipolitics’, whereby the noisy to‐and‐fro of debate and disagreement would be stilled in favour of metaphysical contemplation.^35^ This longing for order has ever since animated the schemes of rationalists seeking to subsume politics to some philosophy or other. An opposed formation, associated with Aristotle, favours republican forms of government, to which argument and, thus, rhetoric are central. Accordingly, not every dispute can or should be solved by rigorous philosophical logic.^36^ Innovation and improvisation in responding to unforeseen situations are important values which are realized through context‐bound deliberation. More than this, if life in community is the ultimate end of the person, then public debate about the welfare of the community is the highest calling upon its citizens and the capacity to speak and be listened to is the key token of their membership of the political community.^37^

Rhetoric, then, is much more than flattery and crowd pleasing; it is the very substance of democratic life, at least as understood in classical Athens (a limitation to which we will return). Correlatively, the stifling of debate constitutes a threat to the liberty not only of individuals, but of the whole polis.^38^ Republican revivals in Renaissance and Enlightenment Europe, and at the inception of the United States of America, were marked by a revalorization of public speech in these terms.^39^ Against the twentieth century understanding of politics as domination, Hannah Arendt saw it as a public space where people articulate and clarify common concerns, though from different points of view.^40^ In this dispensation, who can speak, when, and about what, was and is a matter of constitutional significance. Rhetoric is intimately concerned with these questions. But, as we have seen, it poses them, not primarily in terms of abstract principle, but with reference to concrete verbal and textual practices and the legal ‘infrastructure’ which sustains them. As such, rhetoric partakes of the ‘artificiality’ which Arendt saw as a necessary and valuable feature of the public realm.^41^ And it is by attending to the artifice of discourse that we can identify an apt mode for studying and critiquing law politically.

## CHALLENGING THE CLASSICS

The modern study of rhetoric dates from the turn of the nineteenth century. A revival centred on the liberal arts colleges of the United States at this time was driven by the anxiety of intellectuals, like John Dewey, regarding mass democracy.^42^ As Matthew Arnold had argued of an industrializing Victorian Britain, the newly enfranchised would have to be inducted into full political citizenship with the help of classical exemplars.^43^ In the more deliberative and less oligarchical culture of the United States, this was ideally provided through an education in public speaking. A traditionalist, neo‐Aristotelian understanding of rhetoric, as both instrumental and edifying, held sway in the communication and composition studies departments of these colleges up to the 1960s when it came under sustained critique.^44^ The pressure of new social movements and the ‘turn to talk’ in social theory made rhetoric the focus of a novel concern with power and inequality. Such challenges to rhetoric took two forms. First, an external critique of the meanings of the revival project as a whole; and second, an internal critique of the way in which hierarchy and exclusion are achieved through rhetorical forms and practices.

The external critique highlights the gendered, raced, and classed nature of the idea of rhetoric, and the classics more broadly, as reproduced within European politics and scholarship. Feminist critics note how neo‐Aristotelians take as their privileged object of analysis speech situations from which women were excluded: the Athenian agora, the Roman forum, and, until recently, courtrooms and parliamentary chambers.^45^ The origin story of rhetoric exalts a masculine ideal, where speech is the instrument of dutiful and virtuous democrats, who woo and seduce their audiences by appealing to simple common sense, not philosophical abstraction.^46^ This affirmation of rhetorical virility has long been made in the face of sceptics who castigate rhetoric in feminine terms as the ‘Harlot of the Arts’ or their mere ‘handmaiden’.^47^ The idealized scene of rhetoric was also white and upper‐class, taught and practiced within neo‐classical courthouses, national assemblies, settler mansions, and country houses. The citizenry called to debate and decide on the good of the commonwealth was narrowly defined, excluding slaves and other labourers as had been the case at Athens and Rome.^48^ Rancière calls this Aristotelian conception ‘parapolitics’, broader than the ‘archipolitics’ of Plato's philosopher kings, but still clearly limited.^49^ In Britain today, as Martin and Finlayson note, the prestige rhetorical forms which dominate in parliament, the courts, and the quality media, are most likely to be acquired at fee‐paying schools and in the debating societies of elite universities.^50^ Raka Shome has noted that these were also fora where ‘imperial voices were primarily heard and imperial policies articulated’.^51^ The speech practices of the subaltern largely went undocumented and form no part of the inherited rhetorical canon.^52^ Revivalists, she argues, have been slow to reflect on this exclusive legacy and its contribution to the disciplinary authority (*ethos*) of modern studies in rhetoric.^53^

The internal critique of rhetoric challenged technical conceptions of speaker, audience, and the relationship between them posited in orthodox theories. In a landmark monograph of 1965, Edwin Black argued that object of any rhetorical transaction on the neo‐Aristotelian model is a straightforward judgement about future, past, or present matters (mapping on to the traditional typology of deliberative, forensic, and commemorative speech).^54^ This presumes a set of stable values common to speaker and audience, which the former appeals to in order to produce the judgement desired. However, as Black notes, neo‐Aristotelianism reaches its limits where such stability is lacking, say in situations of social pluralism or conflict, which are in truth more common.^55^ There, rhetorical transactions aim not so much at the production of judgement, as at the fostering of shared convictions and identities. Emotional appeal is more prominent than reason, and often prior, and it is realized through concrete language, rather than abstract values, through presenting vivid pictures of ‘the world as it is’, rather than through principled argumentation. This breaks open the bounded, dyadic and fairly static model of speaker‐audience relations which underpins the instrumentalist understanding of rhetoric in neo‐Aristotelian accounts. It goes beyond the ‘linguistic management of an audience's common store of values’ and embraces Lionel Trilling's understanding of culture as struggle.^56^

These charges were amplified in the case of law by Peter Goodrich in his critique of Chaim Perelman's new rhetoric, itself a major effort to revive Aristotelian traditions as a mode of studying modern argumentation.^57^ Although gesturing towards ‘the historical and practical human character of reason’, this is premised on a conception of law's audiences which suffers from deficiencies similar to those just discussed.^58^ The legal and extra‐legal publics of law, on Perelman's account, are already constituted, homogeneous communities of value. Their acceptance of law's legitimacy is given. What remains to the rhetorical critic is to study the effective techniques of communication deployed intentionally by legal orators promoting increased adherence to legal values and, thus, seeking to win adherence to specific conclusions. As Goodrich notes, this is methodologically unambitious and politically conservative.^59^ Ultimately the deployment of classical rhetoric on its own as a tool for studying contemporary speech is anachronistic and inadequate to the purposes of socio‐legal scholars. It cannot grasp the dynamics of institutional power and social inequality which give shifting content to legal semantics. ^60^ More is needed.

## CONSTITUTING, EXCLUDING, SILENCING

Our task then is to challenge the given‐ness and presumed unity of the audience, and also the given‐ness and pure intentionality of the speaker. Critics suggest that in doing this we need to accept that rhetoric can have a constitutive force, beyond its straightforwardly persuasive effects. An origin point for this approach is to be found in Wayne Booth's notion of the ‘implied author’, an ideal created over the course of a given text by the physical author through stylistic and other devices.^61^ Edwin Black opened out this insight into a more wide‐ranging theory of rhetorical personae.^62^ As little as the speaker (or first persona), the audience of rhetoric should not be conflated with physical listeners and readers. Rather, stylistic tokens and topics deployed in the discourse implied an ideal auditor (or second persona), which the actual listeners could aspire to become. Like the speaker, the audience is a strategic effect of discourse. Maurice Charland's work provides a good example of this, showing how Quebec separatist leaders in the 1970s did more than simply put the case for independence to their listeners and readers.^63^ Rather they called forth (or ‘interpellated’) the latter as a coherent ‘Peuple Québecois’, drawing on historical narratives of linguistic subordination and discrimination. Support for independence was not then a matter for rational judgement, but rather presumed to be already inherent in the collective and individual identities of those addressed.

This widening of critical focus to include a constitutive dimension, has been proposed for legal rhetoric by James Boyd White. For him lawyers’ speech is an argument not only about reasons and results, but also identities. More than an art of persuasion, it creates and re‐creates its objects by drawing on available discursive resources within and beyond the law. A legal instrument contingently creates ‘speakers, roles, topics and occasions for speech’.^64^ It borrows from its own and neighbouring areas of law to do so, for example, contract models of informed consent in medical law, but also from the wider culture, for example, popular and literary stereotypes of health care users, say.^65^ Boyd White takes the United States Constitution, with its allocation of powers, rights, and duties, as the pre‐eminent example of such a legal‐rhetorical text.^66^ That essentially republican instrument called forth a group of speakers as full members of the political community, while others (for example, African‐Americans) have been directly or indirectly excluded for much of its career. The same is true of legislation, such as the English Mental Capacity Act 2005, and the common law regime which preceded it, which we will be examining in the following sections. For Boyd White, law's central question is ‘what kind of community should we be?’ – a question to be answered by reflecting critically on just this allocation of roles, identities, and rhetorical positions.^67^

The first and second personae of rhetoric are produced affirmatively: this is who I am, this is you are, this is who we are, as lawyers, as fellow nationals, and so on. But the critical turn in rhetorical theory has taken us beyond this reciprocal relationship. In a germinal paper Paul Wander proposed that ideal audiences are constituted as much in negation as in affirmation. A ‘third persona’ may be evoked as ‘the silhouette of the second persona’, helping to define speaker and audience, and the relationship between them.^68^ Wander thus reads Martin Heidegger's lectures on art at the University of Frankfurt in 1936 for the manner in which they define the ‘enemies’ of the Nazi regime by implication, most especially persecuted Jews. These are audiences ‘beyond the claims of morality and the bonds of compassion’.^69^ They may overhear the rhetorical exchange, but they are barred from taking part. Building on this, Diana Cloud identifies as the ‘null persona’ of rhetoric potential speakers who censor themselves, through evasive answers or feigned ignorance in the face of threatened violence or discrimination.^70^ Her theoretical advance emerges out of interviews with African‐American cotton‐mill workers in the Deep South of the United States. Silence she argues, can be fully rhetorical, violating audience expectations and signifying, however obliquely.^71^ Adding the third and null personae to our critical repertoire takes us well away from ‘the warmth of the polis’, as evoked by neo‐classical rhetoricians, and towards a more adequate understanding of speech in pluralistic and conflicted societies, and in the legal practices which sustain them.^72^ In so far as law is rhetorical, it is an argument about these constitutional issues, of inclusion and exclusion, as much as about tactics for persuasion. This rhetorical politics of law is central to the rest of our analysis.

## PERSUADING: MENTAL CAPACITY AND BEST INTERESTS

Mental capacity law governs when a person is treated as being capable of making legally valid decisions and performing legal acts. It establishes mechanisms to fill the legal void when a person is regarded as incapable of making such decisions for themselves, for example, by defining what is in their best interests. The field of English mental capacity law is ripe for rhetorical study. For one thing, criteria for determining mental capacity are relatively open‐ended; assessment is situation‐specific, informed by the bodies of knowledge and the values of those professing expertise in this area.^73^ For another, application of the best‐interests test depends on the weighing of a number of factors, none of which is given lexical priority in the relevant legislation.^74^ As a result, in both cases decision making is subject to significant and obvious indeterminacy in seeming contravention of orthodox rule of law values. As Jonathan Montgomery pointed out, judgments in this area are heavily fact‐based, tending towards concrete description and away from abstract normative elaboration.^75^ The parties, their families, and the expert witnesses involved are commonly described in some detail and their evidence reproduced at length and with care. The idiom is commonly richer, more metaphorical, and more anecdotal than in more technical fields. At most, mental capacity and best‐interests judgments are presented as illustrating broadly expressed values, rather than proceeding in deductive fashion from legal rules or developing new law incrementally.^76^ Increasingly judges seek to convince diverse publics of the fairness of their rulings, and of the legitimacy of courts deciding vital questions concerning vulnerable persons, rather than their next of kin, which seems to be the expectation of majority of the general public.^77^ These two purposes call forth a conspicuous effort, one aimed at persuasion, which can be studied using the tools of classical rhetoric set out above.

The rhetorical history of this area is one of change, though not, it should be added, one of inexorable progress. Subject primarily to common law until the mid‐2000s, judicial decision making on capacity and best interests was initially marked by substantial deference to medical opinion and, as such, formed a consistent part of the wider health law field. The test of negligence in *Bolam* v. *Friern Hospital Management Committee* ^78^ enjoyed dominance across a range of controversial medical issues well beyond its proper field of liability for malpractice. Predicating the lawfulness of procedures on conformity with the views of a ‘responsible body of medical practitioners’, the test effectively subjected issues such as non‐voluntary sterilization and the withdrawal of life‐sustaining treatment from incompetent patients, to the judgement of doctors as to patients’ best interests with very limited external scrutiny.^79^ In effect clinical judgement was adopted as a place (*topos*, topic) to which the indeterminacy of legal decision making on medical matters could be displaced and hopefully concealed.^80^

Within the terms developed by Rancière, ‘Bolamized’ medical law instantiated a Platonic archipolitics, whereby legal argument was stilled by being subsumed to clinical reason. The significant audience for this was accordingly composed of a tightly defined community of senior doctors and judges, sustained by the shared culture of British elites in that period. Liberal and feminist scholars, Ian Kennedy and Sally Sheldon, for example, condemned this ratification of medical paternalism.^81^ The ethical charge for their critique was supplied variously by Kantian and Millian work on individual autonomy.^82^ The right to refuse and (somewhat more problematically) to demand treatment was central to these interventions. In substance they proposed to replace the concrete and distinctively English idiom of the gentleman practitioner and his dependent patient, encompassed by the national project of the NHS, with a normative language, aimed at what Chaim Perelman called a universal audience, unbound by time and place.^83^

But the academic challenge should also be understood in republican terms as a demand for the widening of audiences and permitted speakers in debates on health care. This meta‐rhetorical claim was pressed at all levels and instances, for example, in the case of patients encountering doctors in the clinic, but equally by pro‐choice groups challenging predominantly male authority in parliament, the churches, and the professions.^84^ The proposed widening of speakers and addresses inevitably means a diminution in the range of shared common sense and a fracturing in the community called forth by legal rhetoric. The challenge has met with some, though not complete, success.^85^ Rather than an even transition to rhetorical universalism as per Perelman, we observe a pluralistic landscape. Rights to participate in deliberation are conceded, and then partially withdrawn or qualified. More nuanced presentation of his or her situation may lead to an individual's wishes being denied rather than granted.^86^ Far from tending towards a coherent recognition of rights, the multiplication of audiences often subjects decision making to a confusion of populist voices. We explore each of these phenomena with reference to the career of the Mental Capacity Act 2005 which can be taken as a hinge point in the shift from the old to the new legal rhetoric in this area.

## EMPOWERING: THE MENTAL CAPACITY ACT

The political significance and interpretive horizon of the Mental Capacity Act 2005 (MCA) can be investigated by asking what was its ‘rhetorical situation’? In other words, what issue or ‘exigence’ was it presented as addressing, and how was its manner of doing so made plausible?^87^ The classical notions of *stasis* and *kairos*, which we mentioned above, can help in this.^88^ *Stasis* corresponds to the *quaestio* in Roman law. Accordingly, the issue is narrowed down and defined with reference to certain topics which also suggest how it can be resolved.^89^ In this regard, the aim of the Act as passed is now widely taken to be that of protecting the right of individuals to make decisions about their own lives wherever possible.^90^ However, that understanding of the 2005 Act, as one whose primary aim is to empower, is at odds with earlier iterations of its development. At the inception of the reform process a decade earlier, the Law Commission's chief concern had been with the lack of clear statutory provision for making health and welfare decisions on behalf of ‘incapable’ persons.^91^ This had left caregivers at risk of liability for negligence or even battery if they acted without capable consent. The situational ‘exigence’ calling forth a rhetorical response was, thus, defined first and foremost as a legal lacuna, rather than an ethical deficit. Legislative proposals at this stage certainly included individual autonomy in the topic grid of factors defining the problem, but this was positioned as a competing interest to be balanced against others rather than the central impetus for reform itself.^92^

By contrast, the final draft bill of 2003 was unambiguously presented as an instrument to empower people. It would ensure that if some mishap befell them, they would not be treated as completely incapable and denied all opportunity to make decisions. In so far as they were identifiable, past expressed wishes could influence – or even determine – the decisions to be made on their behalf.^93^ The exigence had, thus, been redefined as one of insufficient respect for the self‐determination of disabled people or anyone who might one day lack capacity. The topic grid was adapted, with autonomy interests now prioritized through presumptions contained in s. 1 of the Act and detailed implementation provisions in the Mental Capacity Act Code of 2007.^94^

The shift in definition can grasped by returning to the notion of *kairos* which suggests that speech is prompted by the demands of a particular political conjuncture, and that it can be judged with the reference to whether it responds aptly and in good time. At a general level the ‘moment’ of the MCA was defined by the New Labour administration which came to power in 1997 and which consistently articulated its ambitions in the temporal idiom of renewal and modernization. Empowerment was a central theme in the government's neo‐liberal approach to social policy, from labour activation strategies to the part‐marketization of public services.^95^ Law reform was headlined by the passage of the Human Rights Act in 1998. These general coordinates were matched by the ascendancy of pro‐autonomy perspectives across relevant sections of the academy, legal practice and, increasingly, the judiciary.^96^ At a very basic level, extant paternalism was condemned as more suitable to an earlier age of deference (the 1950s, say) and so ‘out of time’. As regards public expectations, law was taken to be ‘in the rear and limping a little’, in the words of a frequently quoted dictum from Australia.^97^

It would be wrong, however, to cast the turn to ‘empowerment’ as wholly novel. The common law goes forward looking backwards. Its rhetorical substance is never abruptly amputated. Among the liberal virtues claimed for the MCA are its ‘value‐neutrality’ as regards individual choices, whereby capacity is to be analysed only with reference to how a person makes a decision, rather than the outcome of that decision.^98^ This stance is not wholly novel but, rather, has played a consistent if supplementary role in the history of English lunacy and capacity law.^99^ Thus the Act builds on long‐standing protection for what today are known as ‘unwise decisions’, previously termed ‘eccentricity’, a concept which allowed courts to avoid the harsh consequences of a finding of insanity from which it was sharply distinguished. Thus, in the 1862 case of ‘mad’ Windham, the court refused to find the man involved ‘insane’ – meaning he would be divested of control over his property and person – merely because ‘that he was in the habit of driving a railway engine, and that he had purchased a complete suit like a guard.’^100^ The space for such ‘eccentricity’ is seen as being uniquely available in England. As Lord Reid put it in

We have too often seen freedom disappear in other countries not only by *coups d'état* but by gradual erosion: and often it is the first step that counts. So it would be unwise to make even minor concessions.^101^

‘Location’ of judicial reasoning in that way has continued in the Court of Protection established under the MCA with the frequent citation of Magna Carta and J.S. Mill's *On Liberty*, as if they were quasi‐sources of law.^102^ The rhetorical action in each of these cases consists of more than a bare appeal for adherence to ethical principles. Rather, as Maurice Charland's work on Québecois nationalism suggests, what is attempted here is the construction of an audience already committed to a certain kind of liberalism by virtue of its national identity. Having inquired into the broad political common sense instantiated by the 2005 Act, we now move to a more detailed scale, inquiring this time into the changing rhetorical situation of mental capacity and best interests hearings themselves.

## GOVERNING SPEECH, CONSTITUTING SPEAKERS

We saw above that the critics of neo‐Aristotelian rhetoric challenged its preponderant focus on the relationship between speakers and listeners, writers and readers. They proposed a more thoroughgoing inquiry into the constitution of all parties to the rhetorical exchange. They asked hard questions regarding the exclusion and self‐silencing of some audiences. This newer approach is ‘critical’ in its concern with power and inequality. But it also remains ‘rhetorical’ in its attention to concrete speech situations and the verbal and textual strategies deployed within them. Audiences are called forth or marginalized as a result of the creative work of speakers and writers. Of course, there are significant apparatuses of coercion, particularly associated with law, and congealed patterns (or structures) of meaning and common sense. But these are only ever of political significance as they are more or less successfully performed in specific times and places. The section picks out three key features of the rhetorical situation here and poses the issues as a series of questions: how is P portrayed (or ‘represented’) in judicial opinions and doctrinal figures? Who is addressed by decisionmakers in these cases? Can P speak in the course of such proceedings? Must they?

Before proceeding we should admit that our use of the term ‘P’ here is itself a rhetorical move worthy of critical attention. In judicial texts the letter functions as a cipher to protect the anonymity of the individual at the centre of the case. It may be that this usage contributes to the distancing of those individuals from the decision‐making process in the manner that we discuss in the following sub‐section. Moreover, what P stands for is not settled. It may be ‘patient’, which supports a medicalization of the issues in these cases and has connotations of now unfashionable paternalism. It may be ‘person’, which avoids these connotations.

### 1. *How is P represented?*

Two cases in the late 1980s saw the House of Lords faced with the legal lacuna noted in relation to decision making for people lacking capacity.^103^ The cases concerned proposals to sterilize incompetent adult women. In both, the court's ruling was effectively determined by medical opinion through the imposition of *Bolam* as the test of best interests. This move was made plausible on the basis of how P's situation and P themselves were portrayed (or ‘represented’, a productively ambivalent term which we use hereafter). Thus, the exigence justifying irreversible sterilization – most notably, uncontrolled sexual appetite and an inability to practice contraception consistently – proceeded from P's flawed and vulnerable condition.^104^ As classical writers noted, physical performance (*actio*) can make an important contribution to the persuasive effort. But in these cases, P deviated from the ideally self‐contained, able, and male body presumed by law and indeed classical rhetoric. Instead, as Judith Butler's work on the performative nature of gender and sexuality would suggest, their assumed physical state meant they lacked authority (*ethos*) and were disqualified from participating in the process.^105^ Little or no reference was made to their wishes or personal perspective, much less to the deficiencies of social and medical care which rendered them vulnerable to abuse and unwanted pregnancies.^106^

Criticisms of this approach led the Court of Appeal to expand the scope of analysis required.^107^ Accordingly, *Bolam* would establish the range of medically acceptable interventions, but the judge had to make a further choice between them based on social, personal, and emotional factors particular to P. The comprehensive evaluation, according to one influential judgment, would be structured in the tabular form of a balance sheet.^108^ As this two‐dimensional figure suggests, the new regime fell short of producing a nuanced and detailed representation of P.^109^ These doctrinal developments widened the intended audience or ‘second persona’ of judicial rhetoric to include non‐medical, as well as medical professionals. But Ps themselves were still left mute, positioned on the margins of discourse, as third personae, the objects of a more or less reductive representation in terms of clinical medicine or, increasingly, social welfare.

The MCA, which came into force after this series of cases, was widely taken to be challenging such exclusion, as we noted above. Indeed s. 4(6) of the Act expressly lists the wishes and prior expressed values of P among the factors to be taken into account in applying the statutory test of best interests. But has the intended shift in the rhetorical culture been realized? While it is still rare for Ps themselves to challenge an assessment of mental incapacity (or a best‐interests decision), outcome‐oriented tests, which allow their views to be outflanked openly, are now taken to be indefensible. Courts increasingly assert that capacity is a matter to be determined by the judge, who must not simply defer to expert testimony.^110^ By the same measure and in contrast with the earlier cases, a detailed character‐sketch of P is increasingly offered in such decisions.

The 2015 Court of Protection decision in *Kings College NHSFT* v. *C* ^111^ to uphold a patient's refusal of life‐sustaining haemodialysis showcases tensions in this new regime. At first glance MacDonald J's decision was in many way an orthodox performance of judicial liberalism: starting from commonplaces like Lord Donaldson's affirmation in *Re T* (*Adult: Refusal of Treatment*)^112^ that P was entitled to refuse medical treatment for any reason or none at all; setting out verbatim relevant sections from the MCA, an earnest of fidelity to the legal text as original source of his ruling (*discovery*); and positioning himself as a careful decision maker (*ethos*), by stating that the case was ‘difficult’ and ‘finely balanced’.^113^ However, the judge's cool self‐representation contrasted sharply with his rendering of C's life as a tale of decline into ill health and indignity after a season of high living. He depicted her circumstances in vivid, almost visual terms, drawing on quotations from C herself.^114^ The appurtenances of her former life were listed – for example, four husbands, a taste for *Veuve Cliquot* champagne – along with an enduring desire to avoid becoming fat and being unable to wear a bikini, and her present wish ‘to go out with a bang’.^115^ The inevitable connotation here was one of superficiality manifest (again!) in P's body. Indeed, C's expressed desire only to live a life that ‘sparkles’ provided a nickname for the case in the media. Though her refusal of treatment was upheld, MacDonald J's fuller portrait of C ironically draws us away from liberal neutrality and abstraction. Rather, in its dense narrative quality it takes the form of a parable, pointing to the wages of frivolity and warning of moral consequences beyond the causal understanding of biomedicine.^116^ To the extent that the mode of representation (judgmental) cuts against the substantive outcome (neutral) two incompatible communities (one moralistic, one liberal) are called forth by the text. All the while effective rhetorical capacity is still denied to P.^117^

### 2. *Who is addressed?*

We have seen that P is commonly spoken *about* in judicial decisions. But to what extent are they directly spoken to? To a significant degree the range of addressees can be inferred from the style and language of judgments under the MCA. The great majority of these are cast in dense and technical terms unlikely to be intelligible to most Ps unaided or their families, suggesting that fellow lawyers and health care and social work professionals are the ideal auditors of judicial speech in the first instance. On occasion, it is true, judgments include content that is specifically directed to P, as with these comments from Mr Justice Peter Jackson, as he then was:

I hope that you will be happy when you return home. If you accept the support you will be getting from district nurses and carers it may be possible for you to stay there. If you do not accept that support you will probably have to return to a care home.^118^

The same judge broke further ground in a recent family court ruling by writing an entire judgment in simple English intended to be accessible to parents with learning disabilities.^119^ Both of the foregoing were laudable initiatives, but they remain marginal. Thus, the direct address to P was carefully framed as a digression, suggesting by contradistinction that the rest of the judgment was *not* intended to be read by them. Equally, a study of 251 health and welfare files in the Court of Protection identified only one case where the judge had written an explanatory letter to the subject of the case to explain their decision, much less draft a whole judgment in comprehensible language.^120^ In fact there are considerably more examples of Ps being expressly *excluded* from learning the content of judgments concerning them, or the fact they have been given in the first place. Orders have been made, for example, directing that nobody should communicate to P that the media was allowed to report on their case or to bring such reports to their attention, or to inform them that they would be subject to a court‐ordered caesarean section and sterilization.^121^ It is possible that the judgments were to be read by the parties *after* the procedures had been carried out but, again, the elaborate legalistic language in which they were cast suggests that that was not the intention of the court. Even more than in cases of inadequate representation like *Kings College NHSFT* v. C, the denial of rhetorical capacity here is forceful and direct, and it corresponds to the dramatic outcomes imposed.

While the inclusion of Ps as addressees has been uneven, a steadier widening is evident as regards the media. Newspapers have long focused critically on legal developments in this area. Most vocal among them has been the *Daily Mail* which early on cast the MCA as part of ‘a culture war’, promoted by ‘legal commissars’ and aimed at ‘subverting family values’.^122^ State‐backed professionals intervene between Ps and their next of kin; the Court of Protection materializes as ‘the judge in the living room’; and an ordinary ‘gran’ can be jailed for hugging her granddaughter.^123^ These interventions are clearly anti‐state – condemned as ‘the coldest of cold monsters’ by one *Sunday Times* columnist – and structured by the antinomy between England (homely and commonsensical) and continental Europe (authoritarian and ideology‐driven).^124^ This hostility was originally compounded by the rule that hearings should generally be held in private given their intimate subject matter.^125^ Repeated complaints in the media that the Court of Protection was operating as a ‘secret court’ has registered with senior judges, who wonder aloud whether they operate with the ‘consent’ of society.^126^ In response, the past ten years has seen a move towards greater ‘transparency’, with larger numbers of Court of Protection judgments being published, and most health and welfare hearings open to the media and the general public, albeit subject to routine court orders prohibiting the communication of identifying or other restricted information.^127^ The audience for decision‐making has thus widened. Whereas formerly the media merely overheard judicial rhetoric, now they are directly addressed, moving from third to second persona. Moreover, they occupy a relatively privileged position channelling the views of society and helping to constitute it as that audience which is the source of the Court's legitimacy.

### 3. *Who speaks?*

Concerns regarding inadequate representation and limited address may be at least partly met, if P is allowed to participate directly in capacity and best interests proceedings. Notwithstanding its timely orientation to individual autonomy, however, no such provision was included in the MCA or in the Court of Protection Rules 2007 as initially drafted when the Act came into force. Although P is usually joined as a party, they are almost inevitably found to lack litigation capacity and are represented by a litigation friend, who advances the case based on their view of P's best interests. Moreover, until recently judges exercising jurisdiction over health and welfare matters only rarely held personal meetings with Ps or sought evidence from them.^128^ Impetus for change came from the European Court of Human Rights which, in a 2008 ruling, required judges in legal capacity proceedings to have actual contact with the individuals concerned.^129^ In 2015 the Court of Protection Rules were accordingly amended to require judges to facilitate such engagement.^130^ It is unclear how far this has changed existing practice and how frequently Ps now participate in health and welfare proceedings. High‐profile cases such as *Wye Valley NHS Trust* v. *B*, where a meeting with P enabled the court to ‘to get a deeper understanding of [his] personality’, have foregrounded the importance of this.^131^ Unfortunately, however, the Court of Protection's infrastructure – both physical, administrative, and legal – is not geared towards the routine participation of people who may have complex physical, cognitive, psychosocial or health needs.^132^

It is clear then, as a matter of law at least, that Ps may speak and that the court should facilitate this.^133^ But can they be obliged to do so? Case law on this is uneven. In 2011 the Court of Protection held that refusing to submit to a capacity assessment is not of itself evidence of incapacity.^134^ By contrast in 2014 it endorsed the forcible removal of an adult woman with autism spectrum disorder from her home for education and capacity assessment regarding contraception.^135^ Here we encounter a paradox: the empowering ambitions of the MCA may depend on actual or threatened force. Even short of that extreme situation, as Derek Morgan and Kenneth Veitch argued, P's rhetorical performance in consultations may determine the outcome of the capacity test, particularly where he or she is refusing life‐sustaining treatment.^136^ Notwithstanding the formal separation of the evaluation of capacity from best interests, and the presumption in favour of capacity in s. 1(2) of the Act, it is to be feared that patients will come under pressure to argue coherently with reference to their own biography and values, in order to be found competent to refuse. As Marinos Diamantides put it, they must display ‘a proactive attitude toward their suffering [t]hey must speak of it’, thereby reassuring the court and helping it in turn to persuade its own diverse audiences within and beyond the legal system.^137^ Lord Donaldson's dictum in *Re T*, mentioned above, that a patient need not give any reasons for refusing treatment is, thus, not always borne out in practice. Instead, the capacity test is itself a further rhetorical situation, one in which P is called forth as speaker and in which silence or near silence may count against him or her.

## CONCLUSION

This essay is offered as an introduction (or re‐introduction) to rhetoric for socio‐legal scholars and a brief demonstration of its potential in the specific context of mental capacity law. Our aim has been to show that rhetorical categories provide a useful framework for analysing legal communications in detail, and for clarifying difficult questions regarding inequality, respect, and participation. We have showcased both classical and critical approaches in doing so. Classical rhetoric draws our attention to specific interventions and the terms in which they are cast. It is an instrument of what Michael Calvin McGee called ‘cultural surgery’, revealing the common sense, the collectively produced emotions, and the discursive forms of authority which are dominant in a given era, or in a given discipline (like law) and its sub‐disciplines.^138^ We saw that the case law on the rights of people with disabilities in England and Wales was a site for the reproduction and transformation of cultural materials in this way. Rhetoric, thus, offers a means of connecting law with its social contexts without sacrificing nuance and without overlooking the agency of diverse legal actors.

Critical rhetoric invites us to consider what Séamus Heaney called ‘government of the tongue’: the ethics of speech situations in pluralistic and antagonistic societies, quite different from the bounded and stable scene denoted by the Athenian agora and the Roman forum.^139^ More than simply pointing out the fact that certain groups are marginalized or silenced, however, critics concern themselves with how this is realized tactically in discourse, and with how it is challenged. Law provides the infrastructure for these moves and countermoves. Statutory provisions and court procedure, as well as the practice of individual judges, shape the ability of people, such as those with disabilities, to speak effectively of their condition and of their wishes. They are included (or excluded) as addressees along with others such as the media and members of various professions. Changing these rules is a key stake in the politics of mental capacity and in many other fields.

Rhetoric in both classical and critical modes can help us to refurbish the republican politics of speech favoured by renaissance thinkers and revived by Hannah Arendt, among others, in the twentieth century. On this view the ability to participate in debate and deliberation, or what we might call rhetorical capacity, is an index of one's inclusion in the public sphere and, thus, a mark of citizenship. That sphere includes the law courts and any other official or non‐official forum in which rights and interests are decided. Even the most intimate matters – of life and death, sexual expression, and human reproduction – are made public once they come within the purview of the law.^140^ Failures of address and limitations on speaking rights in such cases, and more generally, amount to a denial of rhetorical capacity in law and an abrogation of effective citizenship.

*(This article is the second in a series introducing the reader to methods and theories relevant to advancing socio‐legal research. They are written for the curious rather than the expert reader and provide illustrations of how the theories, methods, and frameworks have been employed and might be used in your work.)*

## Footnotes

1 G. Watt, ‘Touching the Matter of Medical Law’ (2018) 12 *Law and Humanities* 102, at 103.

2 Plato, *Gorgias* (1979) 463b.

3 Aristotle, *The Art of Rhetoric* (1991); Cicero, *On the Ideal Orator* (2001); Quintillian, *The Orator's Education, 5 vols*. (2002).

4 P. Dixon, *Rhetoric* (1971) 42.

5 A. Kronman, ‘Rhetoric’ (1998–9) 67 *University of Cinncinnati Law Rev*. 677, at 684–6.

6 J. Locke, *An Essay Concerning Human Understanding* (1979) 508.

7 S. Fish, *Doing What Comes Naturally: Change, Rhetoric, and the Practice of Theory in Literary and Legal Studies* (1990) 471.

8 See P. Goodrich, ‘Critical Legal Studies in England: Prospective Histories’ (1992) 12 *Oxford J. of Legal Studies* 196, at 212.

9 J.B. White, ‘Law as Rhetoric, Rhetoric as Law. The Arts of Cultural and Communal Life’ (1985) 52 *University of Chicago Law Rev*. 684.

10 P. Goodrich, *Legal Discourse: Studies in Linguistics, Rhetoric and Legal Analysis* (1987) 32–4.

11 G. Wetlaufer, ‘Rhetoric and its Denial in Legal Discourse’ (1990) 76 *Virginia Law Rev*. 1545, at 1572.

12 P. Goodrich, ‘Law and Language’ (1984) 11 *J. of Law and Society* 173, at 185.

13 P. Goodrich, *Reading the Law. A Critical Introduction to Legal Method and Techniques* (1986) 185.

14 Wetlaufer, op. cit., n. 11, pp. 1577 ff.

15 A. Finlayson, ‘Political Science, Political Ideas and Rhetoric’ (2004) 33 *Economy and Society* 528, at 541.

16 For an introduction, see N. Fairclough, *Language and Power* (2015, 3rd edn.).

17 A. Finlayson and J. Martin, ‘Introduction: Rhetoric and the British Way of Politics’ in *Rhetoric in British Politics and Society*, eds. J. Atkins et al. (2014) 11.

18 Q. Skinner, *Visions of Politics. Vol. 1: Regarding Method* (2002) 177.

19 For a contemporary application of these paired concepts, see D.S. Moon, ‘Rhetoric and Devolution – Time and Space in Welsh Labour Rhetoric’ in Atkins et al. (eds.), op. cit., n. 17, p. 87. We discuss them further in a subsequent section.

20 See, generally, M. Jørgensen and L. Phillips, *Discourse Analysis as Theory and Method* (2002).

21 See the useful material referenced in R. Toye, *Rhetoric: A Very Short Introduction* (2013) 114.

22 Aristotle, op. cit. n. 3, p. 75.

23 J.M. Balkin, ‘A Night in the Topics: The Reason of Legal Rhetoric and the Rhetoric of Legal Reason’ in *Law's Stories. Narrative and Rhetoric in the Law*, eds. P. Brooks and P. Gewirtz (1996) 211.

24 R. Barthes, *The Semiotic Challenge* (1994) 75.

25 Goodrich, op. cit., n. 13, p. 192.

26 Barthes, op. cit., n. 24, p. 74.

27 See N. Luhmann, *Das Recht der Gesellschaft* (1993) 165 ff.

28 J. Harrington, *Towards a Rhetoric of Medical Law* (2017) ch. 2; see, further, M. King and C. Thornhill, *Niklas Luhmann's Theory of Politics and Law* (2003) 48.

29 G. Watt, ‘The Art of Advocacy: Renaissance of Rhetoric in the Law School’ (2018) 12 *Law and Humanities* 116.

30 A.W.B. Simpson, ‘The Common Law and Legal Theory’ in *Oxford Essays in Jurisprudence (Second Series)*, ed. A.W.B. Simpson (1973) 99.

31 S. Levinson, ‘The Rhetoric of the Judicial Opinion’ in Brooks and Gewirtz (eds.), op. cit., n. 23, p. 187.

32 S. Levinson and J.M. Balkin, ‘Law, Music, and Other Performing Arts’ (1991) 139 *University of Pennsylvania Law Rev*. 1597; S. Ramshaw, ‘The Paradox of Performative Immediacy: Law, Music, Improvisation’ (2013) 12 *Law, Culture and the Humanities* 6.

33 A. Hutchinson, *It's All in the Game. A Non‐Foundationalist Account of Law and Adjudication* (2000) 193.

34 Plato, *Protagoras* (1996) 356d‐e.

35 J. Rancière, *Disagreement. Politics and Philosophy* (1999) 70.

36 Kronman, op. cit., n. 5, p. 692.

37 H. Arendt, *The Human Condition* (1959) 53.

38 Q. Skinner, *Liberty before Liberalism* (1997).

39 J. Martin, *Politics and Rhetoric. A Critical Introduction* (2014) 26.

40 H. Arendt, *The Portable Hannah Arendt*, ed. P. Baehr (2000) 109.

41 Arendt, op. cit., n. 37, p. 31.

42 M.J. Porrovecchio, ‘Introduction’ in *Contemporary Rhetorical Theory: A Reader*, eds. M.J. Porrovecchio and C.M. Condit (2016, 2nd edn.) 5 ff.

43 See R. Williams, *Culture and Society 1780–1950* (1958) ch. 6.

44 D.P. Gaonkar, ‘The Revival of Rhetoric, the New Rhetoric, and the Rhetorical Turn: Some Distinctions’ (1993) 15 *Informal Logic* 53.

45 M. Brody, *Manly Writing: Gender, Rhetoric and the Rise of Composition* (1993).

46 S.K. Foss and C.L. Griffin, ‘Beyond Persuasion. A Proposal for and Invitational Rhetoric’ (1995) 62 *Communication Monographs* 2; M. Beard, ‘The Public Voice of Women’ *London Rev. of Books*, 20 March 2014, 11.

47 Porrovecchio, op. cit., n. 42, p. 4.

48 See, for example, the discussion of Cicero in M. Beard, *SPQR: A History of Ancient Rome* (2016) 21 ff.

49 Rancière, op. cit., n. 35, p. 72.

50 Finlayson and Martin, op. cit., n. 17, p. 2.

51 R. Shome, ‘Postcolonial Interventions in the Rhetorical Canon: An “Other” View’ (1996) 6 *Communication Theory* 40, at 50.

52 The work of retrieving this tradition has been done more by social historians archiving sermons, popular ballads, and pamphlets than by scholars of rhetoric: see, for example, the sources used in E. Hobsbawm, *Bandits* (2000) and E.P. Thompson, *The Making of the English Working Class* (1963).

53 Shome, op. cit., n. 51, p. 49.

54 E. Black, *Rhetorical Criticism. A Study in Method* (1965) 33.

55 id., p. 126.

56 id., p. 125; L. Trilling, ‘Reality in America’ in *Literary Opinion in America*, ed. M.D. Zabel (1962, 3rd edn.) 408.

57 For a concise introduction, see C. Perelman, *The Realm of Rhetoric* (1982).

58 Goodrich, op. cit., n. 10, pp. 98, 116.

59 id., p. 111.

60 id., p. 124.

61 W. Booth, *The Rhetoric of Fiction* (1983, 2nd edn.) 105.

62 E. Black, ‘The Second Persona’ (1970) 56 *Q. J. of Speech* 109.

63 M. Charland, ‘Constitutive Rhetoric: The Case of the *Peuple Québecois'* (1987) 73 *Q. J. of Speech* 133.

64 J.B. White, *Heracles’ Bow: Essays on the Rhetoric and Poetics of the* Law (1985) 40.

65 A.R. Maclean, *Autonomy, Informed Consent and the Law* (2009) 225–6.

66 White, op. cit., n. 64, p. 41.

67 id., p. 42.

68 P. Wander, ‘The Third Persona. An Ideological Turn in Rhetorical Theory’ (1984) 35 *Central States Speech J*. 197, at 210.

69 id., p. 211.

70 D.L. Cloud, ‘The Null Persona: Race and the Rhetoric of Silence in the Uprising of ‘34’ (1999) 2 *Rhetoric and Public Affairs* 177.

71 id., p. 182.

72 Black, op. cit., n. 62, p. 113.

73 Mental Capacity Act 2005, ss. 2, 3. See, further, F. Freyenhagen and T. O'Shea, ‘Hidden Substance: Mental Disorder as a Challenge to Normatively Neutral Accounts of Autonomy’ (2013) 9 *International J. of Law in Context* 53.

74 Mental Capacity Act 2005, s. 4.

75 J. Montgomery, ‘Rhetoric and “Welfare”’ (1989) 9 *Oxford J. of Legal Studies* 395.

76 See J. Coggon, ‘Intellectual Disability, Autonomy, and Legal Rights’ (2011) 18 *European J. of Health Law* 447.

77 A. Beckett et al., *The Future of Lasting Power of Attorney: Appendices to a Research Report for the Office of the Public Guardian* (2014).

78
*Bolam* v. *Friern Hospital Management Committee* [1957] 1 W.L.R. 582 (McNair J).

79 For a through discussion of the influence of *Bolam* on each of these areas, see J. Miola, *Medical Ethics and Medical Law: A Symbiotic Relationship* (2007).

80 M. Knudsen, ‘Structural Couplings Between Organizations and Function Systems: Looking at Standards in Health Care’ (2007) 14 *Cybernetics and Human Knowing* 111, at 117. The argument here and in the next two paragraphs draws on Harrington, op. cit., n. 28.

81 I. Kennedy, *Treat me Right: Essays in Medical Law and Ethics* (1988); S. Sheldon, *Beyond Control: Medical Power and Abortion Law* (1997).

82 For a comprehensive review, see Maclean, op. cit., n. 65.

83 Perelman, op. cit., n. 57, p. 15.

84 D. Wilson, *The Making of British Bioethics* (2014).

85 See J. Montgomery, ‘Law and the Demoralisation of Medicine’ (2006) 26 *Legal Studies* 185.

86 See, for example, *Royal Borough of Greenwich* v. *CDM* [2018] EWCOP 15 (per Cohen J at para. 28).

87 Writers define the rhetorical situation in realist or constructivist terms: see, respectively, L.F. Bitzer, ‘The Rhetorical Situation’ (1968) 1 *Philosophy and Rhetoric* 1; R.E. Vatz, “The Myth of the Rhetorical Situation’ (1973) 6 *Philosophy and Rhetoric* 154.

88 M. Carter, ‘*Stasis* and *kairos*: Principles of social construction in classical rhetoric’ (1988) 7 *Rhetoric Rev*. 97.

89 These function as a kind of ‘Gestalt’: Martin, op. cit., n. 39, p. 96.

90 For example, see H.M. Government, *Valuing Every Voice, Respecting Every Right: Making the Case for the Mental Capacity Act. The Government's Response to the House of Lords Select Committee Report on the Mental Capacity Act 2005* (2014; Cm. 8884); and House of Lords Select Committee on the Mental Capacity Act 2005, *Mental Capacity Act 2005: Post‐Legislative Scrutiny* (HL 2013–14 139).

91 Law Commission, *Mentally Incapacitated Adults and Decision‐Making: An Overview* (1991) 5.

92 id., p. 9.

93 Department for Constitutional Affairs (DCA), *Draft Mental Incapacity Bill* (2003; Cm. 5859).

94 See Lord Falconer of Thoroton, ‘Foreword’ to DCA, *Mental Capacity Act Code of Practice* (2007).

95 N. Fairclough, *New Labour, New Language* (2000).

96 K. Veitch, *The Jurisdiction of Medical Law* (2007) 13 ff.

97
*Mount Isa Mines* v. *Pusey* (1970) 125 C.L.R. 383 (High Court of Australia per Windeyer J, at 395).

98 Mental Capacity Act 2005, s. 1(4) states that a person is not treated as lacking mental capacity ‘merely’ because their decisions may be regarded by others as ‘unwise’.

99 See J. Craigie, ‘Capacity, Value Neutrality and the Ability to Consider the Future’ (2013) 9 *International J. of Law in Context* 4.

100 As reported contemporaneously by a solicitor: see *The Great Lunacy Case of Mr W. F. Windham (A Report of the Inquiry into the Sanity of W. F. Windham)* (1862) 199.

101
*S* v. *S, W* v. *Official Solicitor* [1970] 3 All E.R. 107, at 111; [1972] A.C., 24, at 43.

102 See, for example, *Rochdale Metropolitan Borough Council* v. *KW* [2014] EWCOP 45 (per Mostyn J, at para. 14); *Kings College Hospital NHS Foundation Trust* v. *C* [2015] EWCOP 80 (per MacDonald J, at para. 2); *London Borough of Hillingdon* v. *Neary* [2011] EWCOP 1377 (per Jackson J, at para. 23).

103
*Re B. (A Minor) (Wardship: Sterilisation)* [1987] 2 All E.R. 211 (HL); *Re F. (Mental Patient: Sterilisation)* [1990] 2 A.C. 1 (HL).

104 K. Keywood, ‘Than a Woman: Embodiment and Sexual Difference in Medical Law’ (2000) 9 *Social and Legal Studies* 495.

105 J. Butler, *Gender Trouble: Feminism and the Subversion of Identity* (1990). For an exploration in the legal context, see S. Cowan, ‘Looking Back (To)wards the Body: Medicalization and the GRA’ (2009) 18 *Social and Legal Studies* 247.

106 R. Lee and D. Morgan, ‘Sterilisation and Mental Handicap: Sapping the Strength of the State?’ (1988) 15 *J. of Law and Society* 229.

107
*Re S.L. (Adult Patient) (Medical Treatment)* [2000] 2 F.C.R 45 (C.A.); *Re A. (Medical Treatment: Male Sterilisation)* [2000] 1 F.C.R. 193 (C.A.).

108
*Re A. (Medical Treatment: Male Sterilisation)* [2000] 1 F.C.R. 193 (C.A. per Thorpe LJ, at 206).

109 K. Keywood, ‘“I'd Rather Keep him Chaste.” Retelling the Story of Sterilisation. Learning Disability and (Non)Sexed Embodiment’ (2001) 9 *Feminist Legal Studies* 185.

110 A. Ruck Keene, ‘Taking Capacity Seriously? Ten Years of Mental Capacity Disputes before England's Court of Protection’ (2019) 62 *International J. of Law and Psychiatry* 56.

111
*Kings College NHSFT*, op. cit., n. 102.

112
*Re T (Adult: Refusal of Treatment)* [1992] EWCA Civ 18 (per Lord Donaldson MR, at para. 3).

113
*Kings College NHSFT*, op. cit., n. 102, paras. 1, 24, 4.

114 See J.A. Blair, ‘The Rhetoric of Visual Arguments’ in *Defining Visual Rhetorics* eds. CA. Hill and M. Helmers (2004) 41.

115
*Kings College NHSFT*, op. cit., n. 102, paras. 14, 9, 10.

116 On the role of parables in legal reasoning, see M. Del Mar, ‘Exemplarity and Narrativity in the Common Law Tradition’ (2013) 25 *Law and Literature* 390.

117 C's family later expressed regret that they had given candid evidence about their mother's life, saying they were dismayed by the media reporting of the case: *Re V (Out of Hours: Reporting Restriction Order* [2015] EWCOP 8 (per Theis J, at para. 15).

118
*Re M (Best Interests: Deprivation of Liberty)* (Rev 1) [2013] EWHC 3456 (CoP per Jackson J, at para. 10).

119
*Jack (A Child: Care and Placement Orders)* [2018] EWFC B12.

120 L. Series et al., *Welfare Cases in the Court of Protection: A Statistical Overview* (2017).

121 See respectively: *P* v. *Independent Print Ltd*. [2011] EWCA Civ 756; *NHS Trust* v. *FG* [2014] EWCOP 30; *The Mental Health Trust* v. *DD* [2014] EWCOP 11.

122 W. Oddie and J. Torode, ‘Legal Commissars Subverting Family Values’ *Daily Mail*. 1 November 1995.

123 ‘Comment: The Old, the Sick and a Cold and Grasping State’ *Mail on Sunday*, 24 October 2009; B. Hewson, ‘Fancy Having a Judge in Your Living Room?’ *Spiked* 10 September 2012, at <http://www.spiked-online.eom/newsite/article/12853#.W3KjbuhKg2w>; A. Martin, ‘Treated Worse than a Dog’ *Daily Mail*, 31 December 2014.

124 D. Lawson, ‘Parents of Vulnerable Adults Deserve a Voice’ *Sunday Times*, 21 October 2018; J. Moir, ‘Locking up 72‐year‐old Grannies is Simply not British’ *Daily Mail*, 2 January 2014.

125 This is provided for in Practice Direction on Transparency 4C, supplementing Part 4 of the Court of Protection Rules 2017, S.I. no. 1035.

126 S. Doughty et al., ‘Secret Court Jails Gran who Hugged her Granddaughter’ *Daily Mail*, 10 June 2014; J. Ensor, ‘Woman Jailed by “Secret Court” for Taking Father out of Care Home Talks for First Time of her Ordeal’ *Telegraph*, 30 May 2013; M. Hedley, *The Modern Judge: Power, Responsibility and Society's Expectations* (2017).

127 Now provided for in Practice Direction, op. cit., n. 125.

128 Series et al., op. cit., n. 120, p. 60.

129
*Shtukaturov* v. *Russia* [2008] ECHR 223; see, also, *X and* 7 v. *Croatia* [2011] ECHR 1835.

130 The amended provision is now contained in Rule 1.2 of the Court of Protection (Amendment) Rules 2017, S.I. no. 1035.

131
*Wye Valley NHS Trust* v. *B* [2015] EWCOP 60 (per Jackson J, at para. 18). For a contextual review of this trend, see L. Series, ‘The Place of Wishes and Feelings in Best Interests Decisions: *Wye Valley NHS Trust v Mr B'* (2016) 79 *Modern Law Rev*. 1101.

132 L. Series et al., *The Participation of P in Welfare Cases in the Court of Protection* (2017).

133 J. Lindsey, ‘Testimonial Injustice and Vulnerability: A Qualitative Analysis of Participation in the Court of Protection’ (2018) 27 *Social and Legal Studies* 273.

134 Re J [2011] CoPLR Con Vol. 717.

135
*The Mental Health Trust* v. *DD (No 2)* [2014] EWCOP 13. It is worth noting that a subsequent ruling in this matter found that this forcible intervention had been counterproductive as regards the progress made towards securing DD's cooperation: *The Mental Health Trust & Anor* v. *DD (No 3)* [2014] EWCOP 44 (per Cobb J, at para. 5).

136 D. Morgan and K. Veitch, ‘Being Ms B: B, Autonomy and the Nature of Legal Regulation’ (2004) 26 *Sydney Law Rev*. 107.

137 M. Diamantides, *The Ethics of Suffering: Modern Law, Philosophy and Medicine* (2000) 64, quoted in Morgan and Veitch id., p. 120.

138 M.C. McGee, ‘Text, Context, and the Fragmentation of Contemporary Culture’ (1990) 54 *Western J. of Speech Communication* 274.

139 S. Heaney, *Government of the Tongue. The 1986 T.S. Eliot Memorial Lectures and Other Critical Writings* (1989).

140 Morgan and Veitch, op. cit., n. 136.

